# The Genetic and Molecular Analyses of Rare Candidate Germline *BRIP1/FANCJ* Variants Implicated in Hereditary Breast and Ovarian Cancers

**DOI:** 10.3390/ijms27021037

**Published:** 2026-01-20

**Authors:** Wejdan M. Alenezi, Larissa Milano, Caitlin T. Fierheller, Corinne Serruya, Timothée Revil, Kathleen K. Oros, Jeffrey P. Bruce, Dan Spiegelman, Trevor Pugh, Anne-Marie Mes-Masson, Diane Provencher, William D. Foulkes, Zaki El Haffaf, Guy Rouleau, Luigi Bouchard, Celia M. T. Greenwood, Jiannis Ragoussis, Jean-Yves Masson, Patricia N. Tonin

**Affiliations:** 1 Department of Human Genetics, McGill University, Montreal, QC H3A 0C7, Canada; 2 Cancer Research Program, Centre for Translational Biology, The Research Institute of McGill University Health Centre, Montreal, QC H4A 3J1, Canada; 3 Department of Molecular Biology, Medical Biochemistry and Pathology, Laval University Cancer Research Center, Quebec City, QC G1V 0A6, Canada; 4 Genome Stability Laboratory, Oncology Division, CHU de Québec (Hôpital de l’Enfant-Jésus) Research Center, Quebec City, QC G1J 1Z4, Canada; 5 McGill Genome Centre, McGill University, Montreal, QC H3A 0G1, Canada; 6 Lady Davis Institute for Medical Research of the Jewish General Hospital, Montreal, QC H3T 1E2, Canada; 7 Princess Margaret Cancer Centre, University Health Network, Toronto, ON M5G 2C1, Canada; 8 Montreal Neurological Institute, McGill University, Montreal, QC H3A 2B4, Canada; 9 Department of Medical Biophysics, University of Toronto, Toronto, ON M5G 1L7, Canada; 10 Ontario Institute for Cancer Research, Toronto, ON M5G 0A3, Canada; 11 Centre de Recherche du Centre Hospitalier de l’Université de Montréal, Institut du Cancer de Montréal, Montreal, QC H2X 0A9, Canada; 12 Departement of Medicine, Université de Montréal, Montreal, QC H3T 1J4, Canada; 13 Division of Gynecologic Oncology, Université de Montréal, Montreal, QC H2W 1T8, Canada; 14 Division of Medical Genetics, McGill University Health Centre, Montreal, QC H3H 1P3, Canada; 15 Department of Medicine, McGill University, Montreal, QC H4A 3J1, Canada; 16 Gerald Bronfman Department of Oncology, McGill University, Montreal, QC H3A 1G5, Canada; 17 Service de Médecine Génique, Centre Hospitalier de l’Université de Montréal, Montreal, QC H2X 3H8, Canada; 18 Department of Biochemistry and Functional Genomics, Université de Sherbrooke, Sherbrooke, QC J1E 4K8, Canada; 19 Department of Medical Biology, Centres Intégrés Universitaires de Santé et de Services Sociaux du Saguenay-Lac-Saint-Jean, Hôpital Universitaire de Chicoutimi, Saguenay, QC G7H 7K9, Canada; 20 Centre de Recherche du Centre Hospitalier l’Université de Sherbrooke, Sherbrooke, QC J1H 5N4, Canada; 21 Department of Epidemiology, Biostatistics and Occupational Health, McGill University, Montreal, QC H3A 1A2, Canada

**Keywords:** *BRIP1*, *FANCJ*, *BACH1*, ovarian and breast cancer, cancer predisposing gene, French Canadian, genetic drift, mitomycin C sensitivity, cisplatin sensitivity

## Abstract

Five rare variants in *BRIP1/FANCJ*, initially identified in ovarian cancer (OC) or breast cancer (BC) cases by the adult hereditary cancer clinics, were investigated for their candidacy as clinically relevant variants. These variants were investigated genetically in a population exhibiting genetic drift and molecularly assayed for biological impact. Using in silico tools, population-based genetic databases and other resources, three of the five reported *BRIP1* variants were likely to be damaging: c.797C>T; p.Thr266Met, c.2087C>T; p.Pro696Leu and c.2990_2993delCAAA; p.Thr997ArgfsTer61. The carrier frequencies ranged from 0 to 0.7% in ancestry-defined cancer groups comprising 47 OC families, 49 hereditary breast and ovarian cancer syndrome families, 142 hereditary breast cancer syndrome families, 435 sporadic OC cases and 563 sporadic BC cases and 0–0.2% in 1025 population-matched controls. Multiple carriers of the these variants were identified in additional population-matched cancer cases. Of the five reported BRIP1 variants, p.Thr266Met, p.Pro696Leu and c.2990_2993delCAAA; p.Thr997ArgfsTer61, which were predicted to be damaging, conferred cellular sensitivity to mitomycin C and cisplatin unlike p.Ser139Ala and p.Ala406Ser. Collectively, our investigation implicates *BRIP1* c.797C>T; p.Thr266Met, c.2087C>T; p.Pro696Leu and c.2990_2993delCAAA; p.Thr997ArgfsTer61 as deleterious variants in OC and BC.

## 1. Introduction

*BRIP1* has been implicated as a hereditary breast cancer (BC) [1] and ovarian cancer (OC) predisposing gene [2]. *BRIP1* was first reported as a BC predisposing gene in 2006 using a candidate gene approach involving hereditary BC (HBC) syndrome families that were not explained by germline pathogenic or likely pathogenic variants (PV/LPVs) in *BRCA1* or *BRCA2* [1]. Subsequent independent studies revealed no association of loss-of-function (LoF) PV/LPVs in *BRIP1* with BC, and its role in BC risk remains equivocal [3,4,5,6,7,8,9,10,11,12,13,14]. *BRIP1* was proposed as an OC predisposing gene in 2011 by a genome-wide association study of cancer cases and controls, including OC [2]. Subsequent studies consistently supported the association of *BRIP1* PV/LPVs with OC, suggesting that they play a role in conferring increased risk to OC [15,16,17]. Carriers of *BRIP1* PV/LPVs in OC and BC cases are rare [18]. Fewer than 1–5% of familial and sporadic OC or BC cases carry *BRIP1* PV/LPVs, which is significantly lower than the 20–80% of familial or 5–20% of sporadic OC or BC carriers of *BRCA1* or *BRCA2* PV/LPVs, depending on the population studied [3,18,19,20]. While OC and BC cases carrying *BRIP1* PV/LPVs are heterozygous [21], individuals homozygous or compound heterozygous for such variants are associated with the Fanconi anemia (FA) complementation group J (FANCJ), a hereditary bone marrow failure syndrome exhibiting susceptibility to cancer [22,23,24,25,26].

*BRIP1*, also known as *BACH1* (BRCA1-Associated C-Terminal Helicase), was discovered in the context of elucidating the biological function of BRCA1 [27]. BRIP1 and BRCA1 bind via their BRCA1 Carboxy-Terminus (BRCT) domains: one BRCT motif in BRIP1 and two in BRCA1 [27,28]. The BRCT domain in BRIP1 plays a critical role in its interaction with BRCA1 as a complex along with other proteins in mediating double-stranded DNA break repair by the FA and homologous recombination (HR) pathways [21,22,29,30]. PVs in the BRCT domains of BRIP1 were shown to impact the repair of DNA double-strand breaks due to the loss of interaction between BRIP1 and BRCA1 [27,31,32]. BRIP1 has seven highly conserved DNA helicase motifs that are essential for catalytic activity for processing the repair of DNA inter- or intra-strand crosslinks (ICLs) via the FA–HR pathways [22,27,29,30,33,34]. PVs in these helicase motifs have been shown to impact the repair of ICLs due to the loss of BRIP1 catalytic activity [31,32,33,35,36].

In this study, we investigated *BRIP1* variants for their candidacy as clinically relevant variants that were initially identified in BC and OC cases from adult hereditary cancer clinics in Canada. We applied a strategy involving the investigation of French Canadians (FCs) of Quebec, a population known to exhibit unique genetic architecture due to genetic drift [3,37,38,39,40,41]. The genetic analysis of this population has facilitated the characterization of candidate PVs in established or proposed OC and BC predisposing genes [3]. A small number of PV/LPVs in *BRCA1* and *BRCA2* [42,43], and one in *PALB2* [44], *RAD51C* [45] and *RAD51D* [45,46] have been shown to have a higher allele frequency in FC OC or BC cases compared with population-matched controls. *BRIP1* was reported as a cancer predisposing gene based on the investigation of the germline variants in the Icelandic population, a well-documented founder population [47], where *BRIP1* c.2040_2041insTT was reported in 318 OC cases (2.36%) versus 0.41% of population-matched controls [2]. *BRIP1* has not yet been fully investigated in the FC population with only one early study reporting no clinically relevant variants in HBC and hereditary breast and OC (HBOC) syndrome families [48]. We investigated candidate *BRIP1* missense variants using in silico tools selected for their best performance to predict their impact on gene function using a strategy have been applied to investigate rare missense variants in *RAD51C* and *RAD51D* identified in familial FC OC cases [45,46]. We then investigated the carrier frequency of our candidates in genetically defined FC OC and BC cases and control study groups. We relate our findings to germline *BRIP1* variants identified in the Pan-Cancer OC and BC cases from The Cancer Genome Atlas (TCGA) [49] and cancer-free controls from the Genome Aggregation Database (gnomAD) v4.1.0 [50]. As our candidate variants have not been characterized for their biological impact, we assayed cell lines complemented with our variants and wild-type (WT) BRIP1 for cellular sensitivity to mitomycin C (MMC), cisplatin and poly (ADP-ribose) polymerase (PARP) inhibitors. Our genetic and molecular investigation of *BRIP1* variants identified in a clinical context of the FC population facilitated the interpretation of candidate variants that are also relevant in other populations.

## 2. Results

### 2.1. Candidate BRIP1 Variants Identified in FC in Adult Hereditary BC and OC Cancer Clinics

Information concerning three OC and four BC probands, with self-reported FC ancestry, who tested positive for a *BRIP1* variant in medical genetic settings were provided for this study (Table S1). The probands had been subjected to a 23-to-34 gene-panel testing for germline and copy number variants (CNVs), except for two cases where CNV testing was performed for only *BRCA1* and *BRCA2*. Medical genetic reports revealed that the four BC probands from independent families carried a frameshift c.2990_2993delCAAA; p.Thr997ArgfsTer61 or missense variant c.415T>G; p.Ser139Ala in *BRIP1*, and the three OC probands each carried a missense variant c.797C>T; p.Thr266Met, c.1216G>T; p.Ala406Ser or c.2087C>T; p.Pro696Leu in the same gene, as shown in Figure S1. These probands were negative for PV/LPV/VUSs and CNVs for all genes tested by the panels, including in *BRCA1* and *BRCA2.*

All five *BRIP1* variants were re-annotated with Ensembl Variant Effect Predictor (VEP) [51,52] using *BRIP1* MANE Select canonical transcript [53] (Table 1) for further characterization. All variants were rare in the non-Finnish European population as part of gnomAD v4.1.0, with an average MAF of 5 × 10^−5^, which corresponds to carrier frequency between 1 in 11,900 to 1 in 85,300 individuals of the same population. While all four missense variants were predicted to be damaging at the protein level by at least one of the selected in silico tools, none were predicted to affect splicing of *BRIP1* transcripts.

**Figure 1:**
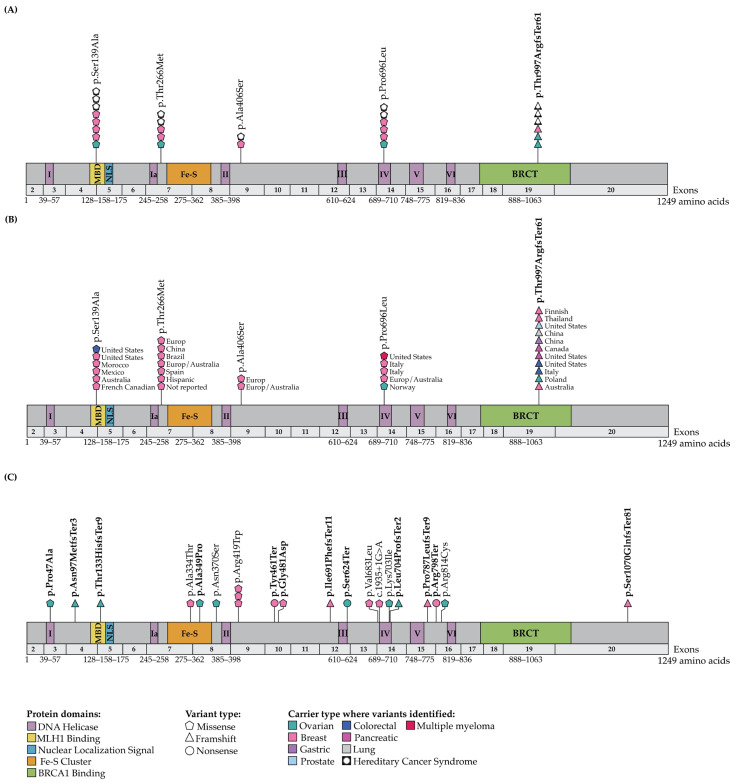
Location of BRIP1 candidate germline variants in the transcript and protein domains. Diagram of *BRIP1* transcript with protein domains indicating the location of candidate variants (see Table S3) identified in the following: (**A**) French Canadian ovarian cancer (OC) and breast cancer (BC) cases in this study; (**B**) different cancers, including OC and BC, reported from a review of the literature; and (**C**) OC and BC Pan-Cancer cases from The Cancer Genome Atlas (TCGA) [49] project, mostly of European origin in this study. *BRIP1* candidate variants classified as pathogenic in ClinVar [54,55,56] or by the American College of Medical Genetics and Genomics guidelines [57] are bolded. Annotation of candidate variants based on the *BRIP1* MANE Select canonical transcript NM_032043.3/ENST00000259008.7 in National Center for Biotechnology Information (NCBI) and European Molecular Biology Laboratory—European Bioinformatics Institute (EMBL-EB.I) [53].

As described in Table 1, c.2990_2993delCAAA; p.Thr997ArgfsTer61, which was reported in two probands diagnosed with BC, is rare in the cancer-free, non-Finnish European population, having an MAF of 4.1 × 10^−5^, and in other populations (Table S2). This variant has been classified as PV/LPV/VUS in ClinVar (Accession number: VCV000234281.23) and PV by ACMG guidelines (Pathogenic Very Strong level 1 [PVS1]; Pathogenic Supporting level 5 [PP5]; and Pathogenic Moderate level 2 [PM2]). This is under the assumption that the encoded protein that has a disrupted BRCT domain that would affect its interaction with BRCA1 as has been reported with *BRIP1* c.2992_2995del; p.Lys998GlufsTer60 [61], another frameshift variant which is located adjacent to our variant. If synthesized, our *BRIP1* frameshift variant is predicted to introduce a premature termination codon at amino acid position 61 and induce truncation of the encoding protein in the BRCT domain (Figure 1A,B). There have been multiple reports in ClinVar of c.2990_2993delCAAA; p.Thr997ArgfsTer61 in the context of hereditary OC, BC as well as FA. The family history of proband PT0152 (Family F1646), carrier of this *BRIP1* frameshift variant was consistent with features of HBC syndrome (Figure S1): an early age of onset of BC (diagnosed between 30 and 39 years of age) and other relatives with an early age of diagnosis with BC, whereas proband PT0164 (Family F1656), who was diagnosed with BC between 30 and 39 years of age and carried the same frameshift variant, had a family history less suggestive of a known hereditary cancer syndrome, though multiple types of cancer were reported in this family.

The four *BRIP1* missense variants of interest were all rare in different populations (Table 1 and Table S2), with MAF of 9.2 × 10^−5^ to 1.2 × 10^−5^ in non-Finnish European controls. The probands PT0147 (Family F1641) and PT0149 (Family F1642) carrying c.415T>G; p.Ser139Ala, were diagnosed with BC between 40 and 49 and 30–39 years of age, respectively (Figure S1). This variant was classified as VUS in ClinVar (VCV000132712.21) in the context of hereditary OC, BC or FA. However, c.415T>G; p.Ser139Ala is classified as likely benign according to ACMG guidelines because multiple in silico tools predict no functional effect on BRIP1 (Benign Supporting 1 and 4 [BP1; and BP4]), shifting its classification toward a likely benign rather than a VUS, while remaining rare in cancer-free controls (Pathogenic Moderate [PM2]). The molecular effect of this amino acid substitution in BRIP1 function is unknown, though it maps within the MLH1 binding domain (MBD) [62] (Figure 1A,B). Interestingly, proband PT0147 (Family F1641) reported a family history of cancer suggestive of Lynch syndrome [63], while proband PT0149 (Family F1642) reported a family history of multiple types of cancer (Figure S1). None of these families had a confirmed case of OC, though there was a second-degree relative of proband PT0147 (Family F1641) suspected of having either uterine cancer or OC. In contrast, the probands carrying the missense variants c.797C>T; p.Thr266Met (PT0150 from Family F1636), c.1216G>T; p.Ala406Ser (PT0151 from Family F1645) or c.2087C>T; p.Pro696Leu (PT0099 from Family F1628) were diagnosed with OC between ages 50–59, 60–69 and 30–39 years of age, respectively (Figure S1). These missense variants were classified as VUSs in the context of hereditary OC, BC or FA in ClinVar (VCV000128196.14; VCV000407821.7; and VCV000128167.23, respectively). Only c.797C>T; p.Thr266Met and c.2087C>T; p.Pro696Leu were classified as VUS by ACMG guidelines because of their rarity (PM2) and both predicted as damaging by multiple in silico tools (PM5). Similarly to c.415T>G; p.Ser139Ala, the c.1216G>T; p.Ala406Ser is classified as likely benign by ACMG guidelines. Although the variant is rare (PM2), multiple in silico tools predict it to be tolerated (BP1, BP4), positioning c.1216G>T; p.Ala406Ser toward a likely benign interpretation rather than a VUS. The molecular effect of these amino acid substitutions in BRIP1 is also unknown, though one maps onto the DNA helicase domain (Figure 1A,B). Though the family history of proband PT0151 (F1645) is suspicious for Lynch syndrome, with two reports of intestinal or colon cancers, the families of all the OC probands carrying these missense variants reported multiple cancer types. There were no striking characteristics of the family history of cancer in probands PT0150 (Family F1636) and PT0099 (Family F1628) (Figure S1).

### 2.2. Multiple Carriers of Candidate BRIP1 Variants Were Identified in Defined FC Cancer Study Groups

The carrier frequencies of *BRIP1* candidate variants were determined in FC study groups comprising familial and sporadic OC or BC cases, regardless of their status of *BRCA1* and *BRCA2* PV/LPVs and in population-matched controls. We did not identify any other carriers of c.2990_2993delCAAA in any of our study groups. However, we identified carriers of each of our missense variants in at least one of our FC study groups (Table 2). We determined that the frequency of carriers ranged from 0 to 0.7% in cancer cases versus 0–0.2% in the population-matched controls, depending on the variant and the group investigated (Table 2). It is interesting to observe that carriers of missense variants were mostly identified in sporadic BC cases, in contrast to OC cases.

It was not possible to determine the carrier frequency of any of the candidate variants in 8493 genotyping-based FC controls (Table S1), as none were represented on the genotyping arrays. However, we were able to impute c.797C>T, as this variant was present in the HRC.r1 haplotype reference panel where no carriers were identified among the genotyping-based controls, suggesting that this variant is rare in the cancer-free FC population.

With our expectation that some of our candidates may occur with a higher frequency in the FC population due to genetic drift [3,39,40], we investigated our candidate *BRIP1* variants in additional cancer cases. We genotyped PBL DNA or surveyed available genetic data of 534 additional OC and 52 sporadic early-onset OC cases for carriers of our candidate *BRIP1* variants. We identified four carriers among the additional group of OC cases, and none in the early-onset cases, carrying c.415T>G; p.Ser139Ala (PT0120), c.1216G>T; p.Ala406Ser (PT0200), c.2087C>T; p.Pro696Leu (PT0119) or c.2990_2993delCAAA; p.Thr997ArgfsTer61 (PT0156) (Table S3). The carrier frequency of each variant in these study groups was less than 2%, which is consistent with the low carrier frequency observed for these variants in the other defined FC study groups (Table 2).

### 2.3. Genetic Analyses of Cancer Cases and Controls Not Selected for FC Ancestry Identified Candidate BRIP1 Variants

Using our criteria for identifying clinically relevant candidate *BRIP1* variants, we investigated genetic data from the germline of 412 OC and 1072 BC Pan-Cancer—TCGA cases and 134,187 cancer-free, non-Finnish European controls. We identified carriers of nine variants in 8/412 (1.9%) OC cases and ten variants in 9/1076 (0.8%) BC cases (Figure 1C and Table S3), which included one BC carrier of c.415T>G; p.Ser139Ala, a candidate variant that was also identified in our FC cases (Table 1). These variants were identified in 0.001–0.09% of the non-cancer, non-Finnish European controls in gnomAD (Table S3). There were 10 LoF (three nonsense, six frameshift and one canonical alternative splicing) and nine missense variants, including c.1109A>G; p.Asn370Ser, that was predicted by SpliceAI [67] to affect splicing that may result in donor loss (Table S3). Eight of these variants map in biologically relevant domains of BRIP1 comprising the MBD and iron-sulfur (Fe-S) [68] and one of the DNA helicase motifs [36] (Figure 1C).

### 2.4. In Cellulo Assays Revealed Deleterious BRIP1 Candidate Variants Affected Cellular Sensitivity to Cisplatin but Not to Olaparib or Talazoparib

**Figure 2:**
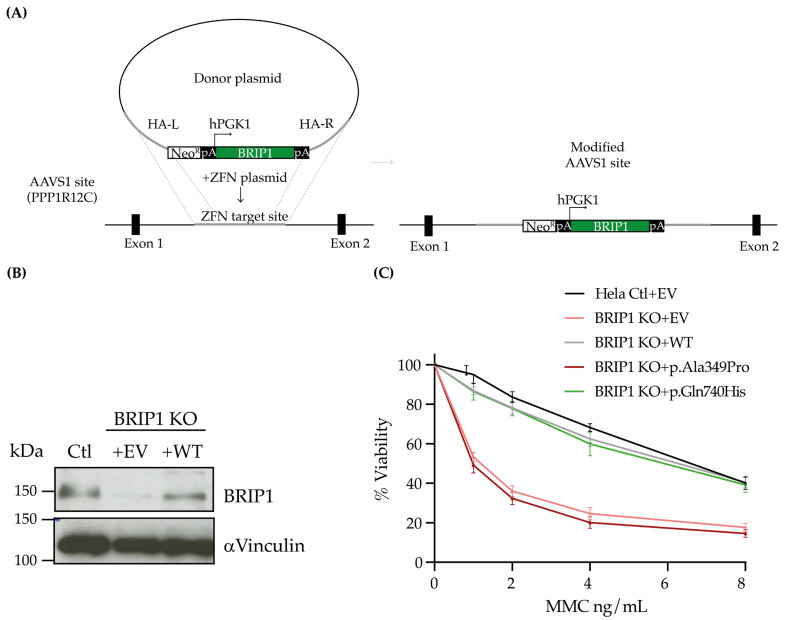
Strategy used to assess the functional impact of BRIP1 variants in a mammalian cell system. (**A**) Scheme representing the AAVS1 genomic editing system used to complement BRIP1 knock-out (KO) cells. (**B**) Hela control (Ctl) cells and BRIP1 KO cells completed with empty vector (EV) or BRIP1 wild-type (WT) using the AAVS1 system were confirmed by Western blot analyses of BRIP1 protein expression, and αvinculin was used as a loading control. (**C**) Survival curves of Hela BRIP1 cells stably complemented with constructs of BRIP1 WT, BRIP1 p.Ala349Pro (a likely pathogenic/pathogenic variant as a positive control), BRIP1 p.Gln740His (a likely benign variant as a negative control) or EV and plated in triplicate in a 96-well plate. Cell viability was monitored following mitomycin (MMC) treatment for 96 h and was assessed by counting remaining nuclei. Experiments were performed in three biological replicates.

To explore the functionality of BRIP1 protein encoded by candidate variants identified in our FC study groups, we generated stable cell lines expressing the following: p.Ser139Ala, p.Thr266Met, p.Ala406Ser, p.Pro696Leu, p.Thr997ArgfsTer61, a BRIP1 WT and an empty vector (EV) using the AAVS1 genomic editing system in BRIP1-depleted cells (Figure 2A,B) [69]. Two BRIP1 variants were included as controls: c.1045G>C; p.Ala349Pro [31,32], which was classified in ClinVar as P/LP (VCV000030535.14) or as a VUS by ACMG and c.2220G>T; p.Gln740His [31,32], which is classified in ClinVar as a VUS or likely benign (VCV000133752.34) or as a VUS by ACMG. BRIP1 p.Ala349Pro was selected as a positive control which was predicted to be damaging at the protein level by all our top selected in silico tools, while BRIP1 p.Gln740His was selected as a negative control which was predicted to be tolerated by all the tools (See Section 4).

BRIP1 activity is critical for mediating the repair of DNA ICLs, and cells deficient for this gene are sensitive to ICL-inducing agents such as MMC and cisplatin [62,70]. Given this phenotype, we assessed the sensitivity of the cells containing the interrogated BRIP1 variants to increasing concentrations of either MMC or cisplatin. As expected, Hela BRIP1 KO cells complemented with the EV were more sensitive to both MMC and cisplatin when compared to the Hela control cells (Ctl) (Figure 2C and Figure S2) [31]. Complementation with the BRIP1 WT rescued the cells sensitivity from the effect of ICL-inducing agents. This phenotype was also observed in BRIP1 KO cells complemented with BRIP1 p.Gln740His, the negative control. However, cells complemented with BRIP1 p.Ala349Pro, the P/LP control, failed to confer resistance to MMC or cisplatin (Figure 2C and Figure S2). A similar profile was observed in cells complemented with the EV. As previously demonstrated [31], greater sensitivity to MMC and cisplatin was also observed in BRIP1-depleted U2OS cells complemented with the EV or BRIP1 p.Ala349Pro, and resistance was partially recovered with the BRIP1 WT (Figure S3).

**Figure 3:**
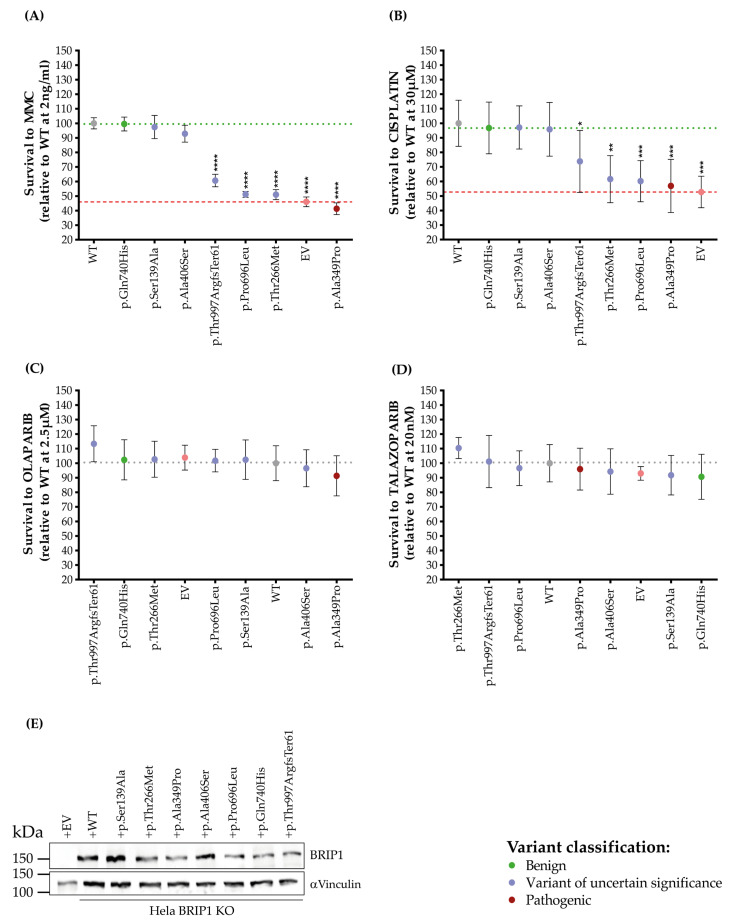
Drug sensitivity of cells expressing BRIP1 variants to DNA inter- and intra-strand crosslinks inducing agents and poly-ADP-ribose polymerase inhibitors. Sensitivity of BRIP1 knock-out (KO) cells stably complemented with constructs of BRIP1 wild-type (WT), BRIP1 variants or empty vector (EV) to the DNA inter- or intra-strand crosslinks (ICLs) inducing agents organized according to survival for each condition: (**A**) Mitomycin C (MMC) and (**B**) cisplatin; and to poly-ADP-ribose polymerase (PARP) inhibitors (**C**) olaparib and (**D**) talazoparib. Sensitivity profiles were determined with the BRIP1 WT set as 100% at the concentrations of 2 ng/mL for MMC (**A**), 30 μM for cisplatin (**B**), 2.5 μM of olaparib (**C**) and 20 nM for talazoparib (**D**). Survival data from each BRIP1 variant is sorted in descending order in response to drug sensitivity and presented based on the mean (±standard error of the mean [SEM]) from at least 3 independent experiments, performed in triplicate. Statistical significance was determined by one-way ANOVA test with Dunnett’s multiple comparison post-test. (*) *p <* 0.05; (**) *p <* 0.01; (***) *p <* 0.001; and (****) *p <* 0.0001. BRIP1 WT is represented in grey, EV in pink, BRIP1 benign variant as a control in green, BRIP1 pathogenic variant as a control in red and BRIP1 variants of uncertain significance in light violet. (**E**) Western blot analysis showing BRIP1 protein expression in BRIP1-depleted cells after stable complementation with the indicated variants, with αvinculin as a loading control.

We next determined whether BRIP1-depleted cells expressing the selected BRIP1 variants were able to confer resistance to the ICL-inducing agents MMC and cisplatin (Figure 3 and Figure S2). BRIP1 p.Ser139Ala and p.Ala406Ser behaved similarly to the BRIP1 WT in terms of the ability to rescue the viability of BRIP1-depleted cells to MMC and to cisplatin, indicating that these missense variants do not impact the functionality of BRIP1 to resolve ICLs (Figure 3A,B). However, cells expressing BRIP1 p.Thr266Met, p.Pro696Leu or p.Thr997ArgfsTer61 were unable to rescue the sensitivity of BRIP1-depleted cells. BRIP1 p.Thr266Met and p.Pro696Leu stood out with the highest sensitivity to the ICL-inducing drugs (Figure 3A,B), with survival percentages each of 51% relative to the WT at a dose of 2 ng/mL of MMC and 61% and 60% relative to WT at a dose of 30 μM of cisplatin, respectively. Using the same criteria, the relative survival of EV was 46% to MMC and 52% to cisplatin, BRIP1 p.Ala349Pro was 41% to MMC and 56% to cisplatin, while BRIP1 p.Thr997ArgfsTer61 sensitivity was 60% and 73% to MMC and cisplatin, respectively (Figure 3A,B). These results provide evidence in favour of the possible impaired function of BRIP1 p.Thr266Met, p.Pro696Leu and p.Thr997ArgfsTer61 variants. Survival curves for all variants are depicted in Figure S2. For further validation, variants were also tested in the U2OS BRIP1-depleted cell line (Figure S3). Similar results to those obtained in Hela cells were observed. However, the dynamic range between U2OS BRIP1-depleted cells complemented with the EV and the WT was proportionately smaller relative to Hela cells. In U2OS cells, BRIP1 p.Ala406Ser appears to have a partial complementation (Figure S3C,I). Concerning protein expression, the BRIP1 variants tested here lead to a protein product, as detected by immunoblotting in our experimental Hela and U2OS cell line models (Figure 3E, Figure S2K and S3K). Considering the impact of PARP inhibitors on the clinical management of BC and OC patients [71], cells expressing our BRIP1 variants were also tested for the sensitivity to PARP inhibitors, olaparib or talazoparib. As previously demonstrated [72], cells depleted in BRIP1 have no greater sensitivity to either olaparib or talazoparib. Complementation with the BRIP1 WT or any of our BRIP1 variants did not alter the resistance profile to either PARP inhibitor (Figure 3C,D and Figure S4).

## 3. Discussion

We investigated five rare *BRIP1* variants that were initially identified in OC or BC cases by the adult hereditary cancer clinics for candidacy as clinically relevant variants. Our genetic analyses of these variants were: (1) assessed bioinformatically their potential impact on gene function; (2) investigated their carrier frequency in defined cancer study groups comprising familial and sporadic OC and BC cases and population-matched controls from a population exhibiting genetic drift; and (3) assessed bioinformatically other candidate variants in *BRIP1* and investigated their carrier frequency in ancestrally diverse cancer study groups and controls. We also assayed biologically the impact of these five *BRIP1* variants on the encoded protein function based on the current known role of BRIP1 in DNA repair [21] and in cellulo sensitivity to chemotherapeutic agents such as MMC and cisplatin and targeted therapeutic agents such as PARP inhibitors [73,74,75]. We also evaluated the protein expression for the different BRIP1 variants. Notably, although the truncated BRIP1 p.Thr997ArgfsTer61 variant migrates at an apparent molecular weight that differs from the expected size, the predicted approximate molecular weight of the 119 kDa truncated product is readily detectable following the immunoprecipitation of FLAG-BRIP1 transfectants (Figure S2K,L). Collectively, our findings from these assays suggest that a frameshift variant c.2990_2993del; p.Thr997ArgfsTer61 and two of the four missense variants c.797C>T; p.Thr266Met and c.2087C>T; p.Pro696Leu likely affect BRIP1 function.

The identification of multiple carriers of each of our *BRIP1* candidate variants is likely attributable to the shared ancestry of the FC population of Quebec [3,41,45,64,76]. We could not determine whether there was a shared genome segment identical-by-descent due to the paucity of carriers of each *BRIP1* variant as *RAD51C* [45], though we have been able to demonstrate shared ancestry using haplotype analyses of the carriers of the most frequently occurring PV/LPVs in *BRCA1*, *BRCA2* [77,78], *PALB2* [44] and *RAD51D* [46] in the context of OC and BC in the FC population [3]. In 2008, an early independent study of *BRIP1* in FC BC cases from HBC or HBOC families of Quebec reported 42 variants in *BRIP1* but concluded that none are likely clinically relevant [48]. We reassessed these variants with our selected in silico tools and retrieved current information from genetic databases (see Table S5), and indeed, none were predicted to be biologically relevant. The only variant found in common with our study of the FC cancer cases was c.415T>G; p.Ser139Ala, which we concur with prior study is a benign variant that is unlikely to affect the protein function. We classified 86% of these reported variants as benign or likely benign based on reports in ClinVar and/or by ACMG guidelines, which is not surprising, as 50% of variants are common, having MAF > 0.01 in the FC controls. The genetic heterogeneity observed in *BRIP1* variant carriers is consistent with the germline genetic landscape of the FC population of Quebec [3]. The differences in carrier frequencies of our variants in *BRIP1* as well as those observed in *BRCA1*, *BRCA2*, *PALB2*, *RAD51C* and *RAD51D* are expected in FCs and consistent with the genetic drift that has been attributed to the waves of localized expansion of this population that occurred in Quebec since 1608 [37,39,41,79,80,81]. Given the European ancestry of FCs, it is not surprising that all five candidate *BRIP1* variants were also identified in the germline of cancer cases in the literature (see Figure 1B). Moreover, the overall low carrier frequency of candidate *BRIP1* variants in FC cancer study groups and the Pan-Cancer—TCGA cases is consistent with the overall low carrier frequency (approximately < 2%) of *BRIP1* PV/LPVs that have been reported in cancer cases from other populations [9,18].

Although the role of our candidate *BRIP1* variants in conferring risk to OC and BC remains to be determined, our in cellulo analyses suggest that they confer an effect on BRIP1 function [82]. BRIP1 binds directly to BRCA1 via BRCT motifs which play a critical role in BRCA1 stability to mediate the repair of double-stranded DNA breaks [15,18,83]. Reports have shown that PV/LPVs effecting the BRCT domain of BRIP1 negatively impact the repair of double-stranded DNA breaks by abrogating the BRIP1-BRCA1 interaction [27], rendering cells sensitive to cisplatin [31,32,36]. We showed that our BRIP1 variant p.Thr997ArgfsTer61, which is predicted to affect the BRCT domain, impaired cellular sensitivity to MMC and cisplatin. Though speculative, this effect may be due to the loss of an intact BRCT domain required for BRCA1 binding. Future studies could include purification of the truncated variant for BRCA1 binding assays as confirmation. Another frameshift variant in *BRIP1* c.2992_2995del; p.Lys998GlufsTer60, which maps to an adjacent amino acid, has also been shown to be expressed in cells [61], suggesting that transcripts from these variants may not elicit nonsense-mediated decay.

Because generating BRIP1 knock-out cell lines in BC or OC models and achieving efficient complementation proved challenging, we used Hela and U2OS cell lines for functional studies where complementation is efficient. Our functional assessment determined that candidate variants p.Thr266Met, p.Pro696Leu and p.Thr997ArgfsTer61 exhibited loss of BRIP1 WT function upon exposure to MMC and cisplatin, while p.Ser139Ala and p.Ala406Ser did not alter cellular sensitivity to these ICL-inducing agents. The proximity of p.Thr266Met and p.Pro696Leu to any one of the helicase domains in BRIP1 may account for the effect on the protein function [31,32] in our assays and warrants further biochemical characterization of helicase activity. Though the lack of BRIP1 results in HR deficiency and loss of replication fork protection, it does not result in PARP inhibitor-induced single-stranded DNA breaks [72,82]. Thus, none of the five variants expressing cells exhibited sensitivity to PARP inhibitors, consistent with independent reports of response to WT and variant BRIP1 response to these inhibitors [64,72,84]. This may have clinical implications for the management of OC and BC patients who are carriers of *BRIP1* PV/LPVs [85,86,87]. Indeed, it has been shown that BC tumour DNA from *BRIP1* carriers did not exhibit a mutational signature characteristic of HR defects, a signature exhibited in *BRCA1* and *BRCA2* carriers exhibiting sensitivity to PARP inhibitors [88]. Although we were able to cultivate BRIP1-deficient cell lines, as also reported by other groups, we had considerable difficulty performing complementation of small interfering RNA (siRNA) BRIP1-deficient cells with a WT construct using a transient transfection system [32]. To overcome this issue, we generated stable cell lines using the AAVS1 system in a CRISPR Cas9 KO background that was able to rescue BRIP1 WT protein. Genomic editing using a donor guide containing the studied variants could be applied to further overcome this barrier.

The bioinformatic tools selected for their best performance [60] to predict the effect of our missense candidate variants on protein function align in part with the results of our MCC and cisplatin sensitivity assays. It is notable that these in silico tools (Table 1) predicted p.Thr266Met and p.Pro696Leu to be damaging, while they predicted p.Ala406Ser and p.Ser139Ala to be tolerated. The prediction scores of the positive and negative controls, p.Ala349Pro and p.Gln740His [31,32], which are classified as LPV and likely benign, respectively, in the ClinVar Database, were consistent with our expectation of these variants as positive and negative controls in our assays. Our observations highlight the relevance of performing functional assays on missense variants, when possible, though this may not be feasible in medical genetics settings.

Due to the small number of carriers in cancer cases, particularly in familial cases, this study was underpowered to address differences in *BRIP1* carrier frequencies in OC versus BC cases in our FC population. It was not feasible to screen all the FC cancer cases investigated in this study for *BRIP1* variants. Though there were more carriers of c.797C>T; p. Thr266Met and c.2087C>T; p.Pro696Leu in sporadic BC cases (5/563, 0.9%) versus sporadic OC cases (1/435, 0.2%), this difference was not statistically different. The low carrier frequency among our HBC families (1/142; 0.7%) was expected, given that *BRIP1* was originally reported as a BC predisposing gene by investigating HBC families [1]. One of the first reports investigating the germline of selected candidate genes involved in the HR pathway in sporadic OC cases reported four carriers of *BRIP1* PV/LPVs, two with a family history of BC [89]. A population-based study investigating genes involved in BC risk reported a statistical difference in carriers of *BRIP1* PV/LPVs in cases with a family history of BC versus controls (20/6361 [0.31%]; odds ratio = 2.15; 95% Confidence Interval [CI]: 1.25 to 3.58); *p* = 0.004) [12], a result consistent with another study [9] and the original report describing *BRIP1* PV/LPVs in familial BC cases [1].

A literature review of our candidate *BRIP1* variants revealed that one of our PV/LPVs, c.2990_2993delCAAA; p.Thr997ArgfsTer61, occurred in the context of hereditary cancers other than BC or OC, such as male BC, cervical, central nervous system, colorectal, head and neck, melanoma, pancreatic, prostate, small cell lung and renal cell-related cancers [63,89,90,91,92,93,94,95,96,97,98,99,100,101,102,103,104,105,106,107,108,109,110,111,112,113,114,115,116,117,118,119,120,121,122] (also see Figure 1B). Carriers of *BRIP1* PV/LPVs have been reported in families with a history of colorectal cancer and other cancer types, or early-onset disease [63,111,112,113,114,115,121,123], that were not explained by known colorectal cancer predisposing genes. *BRIP1* PV/LPVs have also been reported in familial and/or early-onset prostate cancer cases [116,117,118,119,120]. Thus, *BRIP1* PV/LPVs may also be involved in conferring risk to a variety of cancers other than OC or BC.

In conclusion, we applied a strategy to characterize candidate *BRIP1* variants in BC and OC cases that were initially identified in medical genetics settings, providing evidence for their role in hereditary and sporadic disease in a defined population exhibiting genetic drift, and inferred their biological impact applying in cellulo assays. As we have demonstrated in previous studies of other known BC and OC predisposing genes, our strategy in investigating the germline of the genetically unique FC population of Quebec has the potential of identifying variants in cancer predisposing genes that may also be relevant to other populations. Our in cellulo assays involving response to cisplatin and PARP inhibitors revealed the potential impact in abrogating protein function for some of the variants, providing insights on their clinical implications that warrant further investigation in patients carrying *BRIP1* variants. Although penetrance for *BRIP1* variants identified in the FC population has yet to be established, collectively, our findings further support the classification of c.2990_2993del; p.Thr997ArgfsTer61 as pathogenic and provides evidence for the reclassification of c.797C>T; p. Thr266Met and c.2087C>T; p.Pro696Leu from VUS to likely pathogenic missense variants.

## 4. Materials and Methods

### 4.1. Study Groups

The study groups investigated in this report are described in Table S1 [76,124,125,126]. Information concerning *BRIP1* variants in OC or BC cases identified in clinical settings were provided by adult hereditary cancer clinics in Quebec, Canada. Study groups investigated for *BRIP1* variants were from participants selected from the following established biobanks: Banque de tissus et données of the Réseau de recherche sur le cancer of the Fond de recherche du Québec—Santé (RRCancer biobank) (https://www.rrcancer.ca/en/home-2/, last accessed on 1 July 2022); CARTaGENE (https://cartagene.qc.ca, last accessed on 1 July 2022) [45,127]; Université de Sherbrooke—The Genetics of Glucose Regulation in Gestation and Growth (Gen3G) [45,76,124,128]; McGill University—Montreal Neurological Institute (MNI) [45,76,129]; and The Pan-Cancer—TCGA [49]. Clinical data (age of diagnosis, histopathology of cancer, disease stage and tumour grade), genetic reports and family history of cancer from selected cases were provided by the respective biobanks and adult hereditary cancer clinics. Information for each case was anonymized at source. For further protection of anonymity, we assigned a unique identifier (PT with four digits) to each case and modified their respective pedigrees. Criteria for denoting FC ancestry are summarized in Table S1.

### 4.2. Bioinformatic Analyses of BRIP1 Variants Identified in OC or BC Cases of FC Ancestry from Adult Hereditary Cancer Clinics

The *BRIP1* variants in FC OC or BC cases found negative for PV/LPVs in *BRCA1* and *BRCA2* were provided by adult hereditary cancer clinics (Table S1) and were re-annotated using the canonical transcript NM_032043.3 [53]. Variants retained for further analyses were those with a minor allele frequency (MAF) ≤ 0.01 in the general population in gnomAD v4.1.0, and being LoF or missense variants classified as PV, LPV or variants of uncertain of significance (VUSs) in ClinVar [54,55,56] and/or by the American College of Medical Genetics and Genomics (ACMG) guidelines [57]. Missense variants retained for further investigation were those predicted to be conserved and damaging at the RNA or protein level by at least one of the selected in silico tools as described previously [3,45,64,76,124,130,131]. These in silico tools with the cut-off of their scores were selected based on their best performance [59,60,132,133,134]. Splice AI v1.0 [67] with prediction scores of ≥0.5 was applied as an in silico tool predicting effect on the splicing of the transcript, and four tools predicting the effect on protein function were applied: AlphaMissense v1.0 [135], Meta-Predictor of Disease-Causing Variants (Meta-SNP) v1.0 [136], Meta-Recurrent neural network (Meta-RNN) v2.0 [137] and Rare Exome Variant Ensemble Learner (REVEL) v4.0 [138], all with prediction scores of ≥0.7.

### 4.3. Determination of Carrier Frequencies of BRIP1 Variants in Defined FC Cancer and Control Study Groups

The carrier frequencies of our candidate *BRIP1* variants were investigated in FC study groups that have been extensively characterized in previous reports (Table S1). Briefly, the candidate variants were genotyped in peripheral blood lymphocytes (PBLs) DNA from index cancer cases from five different FC groups: 47 OC, 49 HBOC and 142 HBC families [42,43,45,64,76], as well as 435 sporadic OC and 563 sporadic BC cases [45,64,65,76,139], regardless of their status of PV/LPVs in *BRCA1* and *BRCA2.* We genotyped samples using customized TaqMan^®^ [140], Sequenom iPLEX^®^ Gold [141] or Fluidigm^®^ SNP Type^TM^ [142] genotyping assays (primers available upon request), as described previously [45,64,76,130]. Tumour DNA samples were genotyped where PBL DNA was not available. Carrier frequencies of *BRIP1* candidate variants were determined in population-matched controls by surveying 1025 sequencing-based data from the following: 433 from Gen3G, 422 from MNI and 170 from CARTaGENE; and surveying 8493 single nucleotide polymorphism (SNP) genotyping-based controls from CARTaGENE [45,46,64,76,128,143,144]. For probes of variants not presented on the SNP arrays, pre-phasing and imputation were performed using Eagle2 with the Burrows–Wheeler transformation [145] through Sanger Imputation Services (https://www.sanger.ac.uk/tool/sanger-imputation-service/, last accessed on 1 August 2020) using Haplotype Reference Consortium release (HRC.r1) v1.1 as a reference [146], as described previously [45,64,76]. Pair-wise comparisons were performed for carrier frequencies of candidate variants in the different FC cancer groups versus sequencing-based controls. Two-tailed Fisher’s exact test was used to compare carrier frequencies in the cancer versus control groups where un-adjusted *p* values < 0.05 for multiple testing were considered significant.

Additional carriers of candidate variants were identified in OC cases from two resources, as described in Table S1: (1) whole exome sequencing (WES) data was available from 52 sporadic early-onset cancer cases diagnosed with high-grade serous ovarian carcinoma (HGSC) before the age of 50 years [45]; and (2) targeted genotyping of PBL DNA or tumour DNA, as described above from 534 recently recruited OC cases [45,64,66].

Candidate variants were verified in PBL DNA from the identified carriers by bidirectional Sanger sequencing using customized primers (primers available upon request) at the McGill Genome Center, as previously described [45,64,76]. Sequencing chromatograms were visually inspected for variant heterozygosity using 4Peaks v1.8 (https://nucleobytes.com/4peaks/, last accessed on 1 July 2022) (The Netherlands Cancer Institute, Amsterdam, The Netherlands).

### 4.4. Determination of Carrier Frequencies of Candidate BRIP1 Variants Identified in BC and OC Cases and Controls Not Selected for FC Ancestry

We investigated *BRIP1* candidate variants in genetic data from OC and BC cases from the Pan-Cancer—TCGA and non-cancer controls from the gnomAD v4.1.0, and both study groups were not selected for FC ancestry. Variant Call Format (VCF) files that were generated from WES data from the germline of 416 OC and 1072 BC Pan-Cancer—TCGA cases were downloaded as previously described [49,64]. Comma Separated Values (CSVs) files that were generated from WES data from the germline of 134,187 cancer-free, non-Finnish European gnomAD v4.1.0 controls were directly downloaded from gnomad.broadinstitute.org. All variants in *BRIP1* were extracted from these files and annotated as previously described [3,64,76,131]. These variants were subjected to our filtering and prioritizing criteria as described previously [45]. Variants with MAF > 0.01 in the general population in gnomAD v4.1.0 were filtered out, and the remaining variants were prioritized as relevant based on the following: (1) LoF or missense variants predicted to affect splicing by at least 1 out of the 4 in silico tools as described above; (2) classified as PV/LPV in ClinVar and/or by ACMG guidelines; (3) predicted to be conserved by at least one of the three selected in silico tools, as described above; or (4) predicted to be damaging at the level of the protein by at least six of the eight selected in silico tools, as described above.

### 4.5. Generation of Constructs and Cell Lines for in Cellulo Assays of BRIP1 Variants

The pcDNA3-3xFlag-*BRIP1*-WT plasmid, expressing the Human BRIP1 Flag tagged with C-terminal 3X DDK tag, was kindly donated by Bob Brosh (NIA/NIH). The pcDNA3-3xFlag-*BRIP1* constructs harbouring one of our *BRIP1* variants were generated via site-directed mutagenesis using Q5^®^ Site-Directed Mutagenesis Kit (New England Bioloabs, Whitby, ON, Canada) with primers listed in Table S6. The AAVS1 *BRIP1* WT or variant constructs were generated by amplification using the pcDNA3-3xFlag-*BRIP1* plasmids and primers listed in Table S6. Products were cloned into the AAVS1 vector in NotI/PspXI sites [69].

The U2OS (sarcoma derived cell line) and Hela (cervical carcinoma derived cell line) BRIP1 knock-out (KO) and control cells were kindly donated by Sharon Cantor [147,148] and maintained in Dulbecco’s Modified Eagle Medium (DMEM) supplemented with 10% Fetal Bovine Serum (FBS) and 1% Penicillin-Streptomycin. BRIP1 KO cells were stably complemented using the AAVS1 genomic editing system [69]. Briefly, cells were transfected with 4 μg of the AAVS1 construct containing either the WT or one of the BRIP1 variants, along with the 0.4 μg of the pZFN plasmid for 4 h using Lipofectamine 2000 (Invitrogen, Burlington, ON, Canada). After 24 h, transfected cells were selected with Gibco™ Geneticin™ Selective Antibiotic—G418 Sulfate (Thermo Fisher Scientific, Burlington, ON, Canada) for 7 days. Established cell lines containing the BRIP1 variants were maintained in DMEM supplemented with 10% FBS, 1% Penicillin-Streptomycin and 0.5 mg/mL of G418 Sulfate.

### 4.6. Drug Sensitivity Assays

The U2OS or Hela cells were seeded in triplicate assays into a Corning 3603 black-sided clear bottom 96-well microplate (VWR International, LLC, Mississauga, ON, Canada) at a density of 2000 cells per well. MMC, cisplatin and PARP inhibitors (olaparib and talazoparib) sensitivity assays were then performed as previously described [149]. Cells were treated with the indicated drugs for 4 days with concentrations ranging from 0 to 8 ng/mL for MMC, 0 to 60 μM for cisplatin, 0 to 2.5 μM for olaparib and 0 to 40 nM for talazoparib. The entirety of each well was imaged at 4× with Cytation 5 Cell Imaging Multi-Mode Reader (Fisher Scientific, Whitby, ON, Canada) and the Hoechst-stained nuclei were quantified using the Gen5 Data Analysis Software v3.03 (BioTek Instruments, Agilent Technologies, Mississauga, ON, Canada). Cell viability was expressed as a percentage of survival of treated cells relative to vehicle-treated cells. Results represent the mean ± standard error of the mean (SEM) of at least 3 independent experiments, each performed in triplicate.

### 4.7. Protein Extraction and Immunoblotting Assays

Total soluble protein extracts and immunoblotting were performed as previously described [150]. BRIP1 protein expression was detected using a polyclonal antibody (Sigma, #B1310, Sigma, Oakville, ON, Canada). Anti-Tubulin (Abcam, #ab7291, Abcam, Waltham, MA, USA) served as the loading control. Anti-rabbit or anti-mouse IgG (Jackson ImmunoResearch—CEDARLANE Burlington, ON, Canada) conjugated to horseradish peroxidase were used as secondary antibodies.

## Figures and Tables

**Table 1:** Characteristics of *BRIP1* candidate variants identified in carrier probands with ovarian or breast cancer cases of French Canadians from the adult hereditary cancer clinics.

Genomic Features (GRCh38)					
Genome position	17-61849221-A-C	17-61808588-G-A	17-61799224-C-A	17-61776411-G-A	17-61684052-CTTT
Coding change	c.415T>G	c.797C>T	c.1216G>T	c.2087C>T	c.2990_2993delCAAA
Protein change	p.Ser139Ala	p.Thr266Met	p.Ala406Ser	p.Pro696Leu	p.Thr997ArgfsTer61
**Probands**					
OC cases (n = 3)	0	1	1	1	0
BC cases (n = 4)	2	0	0	0	2
**Frequencies in gnomAD ^1^**					
Allele frequency in non-Finnish European (non-Finnish European carriers out of all)	8.3 × 10^−5^ (44/589,661)	1.5 × 10^−5^ (9/589,888)	1.2 × 10^−5^ (7/589,827)	9.2 × 10^−5^ (54/590,000)	4.1 × 10^−5^ (24/589,947)
**Clinical classification ^2^**					
ClinVar (number of submissions contributed to this classification out of the total of submissions; review status out of 5)	VUS (18/21; 2)	VUS (13/16; 2)	VUS (7/7; 2)	VUS (15/18; 2)	Conflicting: P/LP (21/28; 1); VUS (2/28; 1)
ACMG guidelines (implemented rule)	LB (PM2; BP1; BP4)	VUS (PM2; PM5)	LB (PM2; BP1; BP4)	VUS (PM2; BP1)	P (PVS1; PP5; PM2)
**Predictions by in silico tools ^3^**					
AlphaMissense	Benign Moderate	Uncertain	Benign Moderate	Uncertain	-
REVEL	Likely Benign	Possibly Pathogenic	Likely Benign	Highly Pathogenic	-
Meta-SNP	Neutral	Disease Causing	Neutral	Disease Causing	-
Meta-RNN	Damaging	Damaging	Damaging	Damaging	-
Splice AI	-	-	-	-	-

Annotation of candidate variants (see Tables S2 and S3) based on the *BRIP1* MANE Select canonical transcript NM_032043.3/ENST00000259008.7 in National Center for Biotechnology Information (NCBI) and European Molecular Biology Laboratory—European Bioinformatics Institute (EMBL-EBI) [53]. ^1^ Allele frequencies in non-cancer, non-Finnish European controls from the Genome Aggregation Database (gnomAD) v4.1.0 [50]. ^2^ Clinical classifications from ClinVar [54,55,56] based on last revision in Sept 2025, and the American College of Medical Genetics and Genomics (ACMG) guidelines [57,58]. ^3^ Applied predictive in silico tools for conservation and damaging at the protein level selected based on their best performance [59,60]. BC: Breast cancer; BP1: Benign Supporting 1; BP4: Benign Supporting 1; OC: Ovarian cancer; P: Pathogenic; LB: Likely Benign; LP: Likely Pathogenic; PM2: Pathogenic Moderate level 2; PM5: Pathogenic Moderate level 5; PP5: Pathogenic Supporting level 5; PVS1: Pathogenic Very Strong level 1; VUS: Uncertain significance and (-): Not applicable/reported.

**Table 2:** Carrier frequencies of candidate *BRIP1* variants in French Canadian cancer cases and population-matched controls.

*BRIP1* Variant	Study Groups ^1^	Cancer Cases Tested ^1^	Number of Tested Participants (or Families) Per Study Group	Number of Variant Carriers (%)
c.415T>G; p.Ser139Ala	OC families	OC	66 (47)	0
	HBOC families	OC or BC	49 (49)	0
	HBC families	BC	142 (142)	0
	Sporadic OC cases	OC	435	0
	Sporadic BC cases	BC	563	1/563 (0.2)
	FC sequencing-based controls	-	1025	2/1025 (0.2)
c.797C>T; p.Thr266Met	OC families	OC	66 (47)	0
	HBOC families	OC or BC	49 (49)	0
	HBC families	BC	142 (142)	1/142 (0.7)
	Sporadic OC cases	OC	435	1/435 (0.2)
	Sporadic BC cases	BC	563	2/563 (0.4)
	FC sequencing-based controls	-	1025	0
c.1216G>T; p.Ala406Ser	OC families	OC	66 (47)	0
	HBOC families	OC or BC	49 (49)	0
	HBC families	BC	142 (142)	0
	Sporadic OC cases	OC	435	0
	Sporadic BC cases	BC	563	2/563 (0.4)
	FC sequencing-based controls	-	1025	0
c.2087C>T; p.Pro696Leu	OC families	OC	66 (47)	0
	HBOC families	OC or BC	49 (49)	0
	HBC families	BC	142 (142)	0
	Sporadic OC cases	OC	435	0
	Sporadic BC cases	BC	563	1/563 (0.2)
	FC sequencing-based controls	-	1025	0
c.2990_2993delCAAA; p.Thr997ArgfsTer61	OC families	OC	66 (47)	0
	HBOC families	OC or BC	49 (49)	0
	HBC families	BC	142 (142)	0
	Sporadic OC cases	OC	435	0
	Sporadic BC cases	BC	563	0
	FC sequencing-based controls	-	1025	0

The annotation of these variants can be seen in Tables S3 and S4, and the clinic-pathological characteristics of their carriers can be seen in Table S4. ^1^ Study groups comprising ovarian cancer (OC), breast cancer (BC), hereditary breast and ovarian cancer (HBOC) syndrome and hereditary breast cancer (HBC) syndrome families, all from the French Canadian (FC) population of Quebec [3,42,43,45,64,65,66]. Dash (-) denotes not applicable.

## Data Availability

The original contributions presented in this study are included in the article/Supplementary Materials. Further inquiries can be directed to the corresponding author.

## Supplementary Materials

The following supporting information can be downloaded at https://www.mdpi.com/article/10.3390/ijms27021037/s1.
