# Supplementary material for: The Genetic and Molecular Analyses of Rare Candidate Germline BRIP1/FANCJ Variants Implicated in Hereditary Breast and Ovarian Cancers
Source: Int J Mol Sci. 2026 Jan 20;27(2):1037. doi: 10.3390/ijms27021037 (PMC12842359; doi:10.3390/ijms27021037)
Supplement: Supplementary file 1 [file ijms-27-01037-s001.zip › ijms-3939313-18112015-supplementary-figures-13012026.pdf]

Figure S1. Anonymized pedigrees of index ovarian or breast cancer cases carrying a candidate *BRIP1* variant reported by the adult hereditary cancer clinics.
Carrier status of index cases (arrow) tested positive are denoted by plus sign. All carriers were found in a heterozygous state. Age ranges in years is shown with cancer diagnosis or death. Unconfirmed cancer status as reported by the index case was denoted by a question mark (?) beside the reported cancer. All breast cancer (BC) cases were invasive unless stated otherwise (see **Table S5**). Superscript C denotes histological subtype that was confirmed by pathology report.

Figure S2. Sensitivity curves of BRIP1 variants to DNA inter- or intra-strand crosslinks inducing agents in Hela cells.
Survival curves contrasting the abilities of BRIP1 wild-type (WT) and the indicated variants, including the empty vector (EV), to rescue mitomycin C (MMC) (A-E) and cisplatin (F-J) resistance in Hela BRIP1-depleted cells. (K) Western blot representing expression of the indicated variants in Hela BRIP1 depleted cells. (L) BRIP1-FLAG Immunoprecipitation (IP) from stable cell lines expressing each variant from a safe harbour locus.

Figure S3. Sensitivity curves of BRIP1 variants to DNA inter- or intra-strand crosslinks inducing agents in U2OS cells.
Survival curves contrasting the abilities of BRIP1 wild-type (WT) and the indicated variants, including the empty vector (EV), to rescue mitomycin C (MMC) (A-E) and cisplatin (F-J) resistance in U2OS BRIP1-depleted cells. (K) Western blot representing expression of the indicated variants in U2OS BRIP1 depleted cells.

Figure S4. Sensitivity curves of BRIP1 variants to poly (ADP-ribose) polymerase inhibitors in Hela cells.
Survival curves contrasting the abilities of BRIP1 wild-type (WT) and the indicated variants, including the empty vector (EV), to rescue PARP inhibitors: olaparib (A-E) and talazoparib (F-J) resistance in Hela BRIP1-depleted cell
